# The addition of *Lactobacillus* spp. negatively affects *Mycoplasma bovis* viability in bovine cervical mucus

**DOI:** 10.1186/s12917-020-02454-9

**Published:** 2020-07-20

**Authors:** A. García-Galán, C. De la Fe, J. Gomis, E. Bataller, A. Sánchez, J. J. Quereda, E. García-Roselló, A. Gómez-Martín

**Affiliations:** 1 Ruminant Health Research Group, Department of Animal Health, Faculty of Veterinary Sciences, Regional Campus of International Excellence “Campus Mare Nostrum”, University of Murcia, 30100 Murcia, Spain; 2 Microbiological Agents Associated with Reproduction (ProVaginBio) Research Group, Universidad Cardenal Herrera-CEU, CEU Universities, 46113 Valencia, Spain

**Keywords:** In vitro, Viability, *Mycoplasma bovis*, *Lactobacillus* spp., Cervical mucus, Cows

## Abstract

**Background:**

*Mycoplasma bovis* is an important pathogen for the cattle industry worldwide causing significant economic losses. Several transmission routes, including those related to reproduction, have been described. Indeed, the pathogen can colonize the female reproductive tract after artificial insemination (AI) with contaminated semen. *Lactobacillus* spp.-based probiotics have been used for vaginal dysbiosis treatment in women and cows although their role in controlling cervico-vaginal infections due to *M. bovis* is unknown. The objective of the present work is to assess the viability of *M. bovis* (PG45, NCTC 10131) in experimentally contaminated cervical mucus after the addition of *Lactobacillus* spp. at different concentrations as a competing agent and pH acidifier.

**Results:**

The addition of probiotic at a concentration higher than 10^8^ colony forming units (CFU/mL had a detrimental effect (*P* < 0.05) on mycoplasma viability in cervical mucus. This coincided with a significant LAB growth and an important decrease in pH from 8.4 to 5.6 (*P* < 0.05). However, after the addition of less concentrated probiotic, *M. bovis* survival was not affected and there was no significant LAB growth despite the drop of pH from 8.4 to 6.73 (*P* < 0.05).

**Conclusion:**

The addition of concentrations higher than 10^8^ CFU/mL of *Lactobacillus* spp. negatively affects *M. bovis* viability in bovine cervical mucus under in vitro conditions. Although the effect observed on the pathogen viability seems to be related to the pH decrease after LAB proliferation in cervical mucus, further studies are necessary to elucidate if other factors are implicated. Nevertheless, the administration of *Lactobacillus* spp.-based probiotics might be used in the future to control *M. bovis* proliferation in the cervico-vaginal tract of cows.

## Background

*Mycoplasma bovis* is a major etiological agent of bovine mycoplasmosis, widely distributed and responsible for important economic losses hardly quantifiable [1]. The complex epidemiology of the infection frequently complicates the establishment of effective control measures. In this sense, the transmission capacity of *M. bovis* by natural breeding and artificial insemination (AI) has been described, even when using semen extenders supplemented with antimicrobials. The pathogen may cause reproductive disorders such as endometritis, salpingitis, oophoritis, infertility, and abortion [2, 3]. Moreover, semen was the source of *M. bovis* mastitis outbreaks in two closed dairy herds in Finland [4].

In the case of some animals, *M. bovis* has been recovered up to 8 months after insemination in cervico-vaginal mucus [2]. Cervical mucus is a hydrogel secreted in the cervical endothelium and it is composed of water in more than 90% as well as soluble and non-soluble particles [5–7]. Additionally, antimicrobial peptides have been isolated in cervical mucus and they could play a fundamental role in preventing infections [8]. However, the viability of *M. bovis* does not seem to be affected by cervical mucus [2].

As *M. bovis* can survive the entire fertilization process and generate infected embryos [9], many strategies based on the use of antimicrobials have been used to control its presence in semen [10, 11]. However, the in vitro antimicrobial susceptibility of the majority of *M. bovis* circulating strains has declined dramatically in recent years [12, 13]. On the other hand, *Lactobacillus* spp.-based probiotics have been used for vaginal dysbiosis treatment in women as they have shown germicidal action against pathogenic bacteria by competing for resources, stimulating the host immune system, and producing organic acids which decrease pH [14–16]. Lactic acid bacteria (LAB) of the genus *Lactobacillus* are part of the vaginal saprophytic flora of cows although at low abundances [17, 18]. The intravaginal administration of LAB in periparturient cows can modulate the incidence of purulent vaginal discharges, expedite the uterine involution and lower the incidence of uterine infections [19–21]. Nevertheless, the role of LAB in controlling cervico-vaginal infections due to *M. bovis* presence still has to be studied.

Mycoplasmas lack a cell wall and therefore they are very sensitive to external changes such as pH variations [22]. In this sense, several authors have observed the effectiveness of the medium acidification to control the growth of mycoplasmas [23–25]. In cows, the in vivo pH of the cervical mucus is generally about 6.8 and rises to about 7.6 to 8.1 during estrus [5, 26, 27]. These pH values could promote the survival of mycoplasmas whose optimal pH for growing ranges from 7.3 to 7.8 [28]. Therefore, we wonder whether the addition of LAB as competing agents and pH acidifiers to cervical mucus may affect the viability of *M. bovis*.

This work was conducted to assess the viability of *M. bovis* in cervical mucus after the addition of *Lactobacillus* spp.-based probiotic at different concentrations under in vitro conditions.

## Results

Seven in vitro conditions were prepared in cervical mucus and/or modified SP4 to evaluate the viability of *M. bovis* after the addition of different concentrations of *Lactobacillus* spp. (Table 1). The statistical analysis revealed that the analytical condition, the time, and their interaction significantly contributed to the variation in pH, the log colony forming units (CFU)/mL of *M. bovis* and the log CFU/mL of LAB.

**Table 1:** Experimental conditions evaluated

Condition	Composition	*M. bovis*	LAB
1	CM _(1460 μL)_ + MB _(40 μL)_	☑	☑*
2	CM _(1460 μL)_ + L1 _(40 μL)_		☑
3	CM _(1000 μL)_ + L2 _(500 μL)_		☑
4	CM _(1420 μL)_ + MB _(40 μL)_ + L1 _(40 μL)_	☑	☑
5	CM _(960 μL)_ + MB _(40 μL)_ + L2 _(500 μL)_	☑	☑
6	SP4 _(1420 μL)_ + MB _(40 μL)_ + L1 _(40 μL)_	☑	☑
7	SP4 _(960 μL)_ + MB _(40 μL)_ + L2 _(500 μL)_	☑	☑

CM: Cervical mucus. MB: *M. bovis.* SP4: Specific medium for *Mycoplasma* spp. isolation (conditions without CM). L1: *Lactobacillus* spp. at a concentration of 3.24 × 10^6^ UFC/mL. L2: *Lactobacillus* spp. at a concentration of 3.24 × 10^8^ UFC/mL. LAB: Lactic acid bacteria. ☑: Counts of *M. bovis* and lactic acid bacteria were made.* Lactic acid bacteria were isolated in the cervical mucus pool. The experimental conditions were prepared following a protocol previously described [25]

The impact of each analytical condition on *M. bovis* survival, disregarding the time, is shown in Table 2. No differences were found between conditions 1 and 4 (*P* > 0.05). Significant differences were found between the equivalent conditions 1 and 4, and the conditions 5, 6 and 7, which were also statistically different from each other (*P <* 0.05). A detrimental effect (*P* < 0.05) on the mycoplasma viability was observed when adding L2 in cervical mucus (condition 5). However, this effect was not observed after adding L1 (condition 4) being *M. bovis* viability equal than in contaminated cervical mucus without probiotic (condition 1). The addition of probiotic in SP4 (conditions 6 and 7) did not affect the survival of the mycoplasma, whose concentration was higher (*P* < 0.05) than in cervical mucus conditions (1, 4 and 5).

**Table 2:** Least squares means of log CFU/mL of *M. bovis*

Condition	Composition	Log CFU/ml of *M. bovis*^1^
1	CM + MB	7.19^a^
4	CM + MB + L1	7.04^a^
5	CM+ MB *+* L2	3.77^b^
6	SP4 + MB + L1	8.05^c^
7	SP4 + MB *+* L2	7.82^d^

^a, b, c, d^: Means with different superscripts between conditions differ significantly (*P* < 0.05). ^1^Standard error of the mean (SEM): 0.08. CM: cervical mucus. MB: *M. bovis*. SP4: Specific medium for *Mycoplasma* spp. isolation (conditions without CM). L1: *Lactobacillus* spp. at a concentration of 3.24 × 10^6^ UFC/mL. L2: *Lactobacillus* spp. at a concentration of 3.24 × 10^8^ UFC/mL. Conditions not contaminated with mycoplasmas are not included

The impact of the time on the pH, log CFU/mL of *M. bovis* and log CFU/mL of LAB under each condition is shown in Table 3. In cervical mucus without probiotic addition (condition 1), neither significant variation of pH nor *M. bovis* concentration was observed. Under this condition, LAB were quantified, even though neither L1 nor L2 had been added. The inoculums L1 and L2 were viable in cervical mucus and LAB concentration increased over time while pH decreased (*P <* 0.05; Conditions 2 and 3). In any case, the pH decrease was higher when adding L2 (8.40 to 5.60) that when adding L1 (8.4 to 6.64). No significant increase of LAB concentration was observed in contaminated cervical mucus after adding L1 (condition 4) and *M. bovis* grew significantly despite the drop of pH from 8.4 to 6.73 (*P* < 0.05). On the other hand, the LAB grew significantly in contaminated cervical mucus after adding L2 and *M. bovis* viability was negatively affected since no viable colony was counted (*P* < 0.05; Condition 5). That concurred with an important reduction in pH from 8.4 to 5.6 (*P* < 0.05). Finally, neither significant LAB growth nor pH variation was observed in SP4 inoculated with L1 or L2 while *M. bovis* grew significantly (conditions 6 and 7).

**Table 3:** Least squares means of pH and log CFU/mL of *M. bovis* and LAB by time

Condition	Composition	Hour (h)	pH^1^	Log CFU/ml of *M. bovis*^2^	Log CFU/ml of LAB^3^
1	CM + *MB*	0	8.40^a^	7.23^a^	0^a^
		15	7.52^a^	7.16^a^	2.22^a^
2	CM + L1	0	8.40^a^	–	1.81^a^
		15	6.64^b^	–	7.40^b^
3	CM + L2	0	8.40^a^	–	3.84^a^
		15	5.60^b^	–	11.66^b^
4	CM + MB + L1	0	8.40^a^	6.44^a^	2.41^a^
		15	6.73^b^	7.65^b^	7.32^a^
5	CM+ MB *+* L2	0	8.40^a^	7.56^a^	3.20^a^
		15	5.60^b^	0^b^	9.75^b^
6	SP4 + MB + L1	0	7.60^a^	7.38^a^	1.93^a^
		15	7.19^a^	8.72^b^	4.34^a^
7	SP4 + MB *+ L2*	0	7.60^a^	7.45^a^	3.31^a^
		15	7.21^a^	8.19^b^	5.44^a^

^a, b^: Means with different superscripts between the times within conditions differ significantly (*P* < 0.05).^1^Standard error of the mean (SEM): 0.34; ^2^SEM: 0.11; ^3^ SEM: 1.86. CM: cervical mucus. MB: M. bovis. SP4: Specific medium for *Mycoplasma* spp. isolation (conditions without CM). L1: *Lactobacillus* spp. less concentrated (3.24 × 10^6^ UFC/mL). L2: *Lactobacillus* spp. more concentrated (3.24 × 10^8^ UFC/mL). LAB: Lactic acid bacteria

## Discussion

Under in vitro conditions, the survival of *M. bovis* in cervical mucus was affected by the addition of *Lactobacillus* spp.-based probiotic at a concentration of 3.24 × 10^8^ CFU/mL. On the contrary, the addition of 3.24 × 10^6^ CFU/mL of *Lactobacillus* spp. did not have a detrimental effect on mycoplasma survival.

Our results suggest that the negative impact on the pathogen viability may depend on the dose of probiotic added to cervical mucus. Other authors previously observed that bovine vaginal lactobacilli at a dose of 10^8^–10^9^ CFU/mL were able to modulate the incidence of purulent vaginal discharges, expedite the uterine involution and lower the incidence of uterine infections, in periparturient dairy cows [19–21]. On the other hand, L1 was previously inoculated vaginally in an ovine experimental model without producing alterations in the ewes health status and without negatively affecting their fertility, either [29]. It must be stated that the evidence of positive effects from that preliminary study (increases in fertility percentage and decrease in the percentage of vaginal neutrophils) were not significant. Overall, the negative effect on *M. bovis* viability caused by the addition of L2 but not L1, together with the observations of previous studies, suggests that further in vivo studies should consider the inoculation with L2.

It is known that vaginal *Lactobacillus* spp. can inhibit the growth of genitourinary pathogenic microorganisms through different mechanisms such as the production of organic acids that reduce pH [15, 16]. As mycoplasmas do not synthesize the cell wall, they are sensitive to external changes such as pH and osmotic variations [22]. We hypothesized that by adding *Lactobacillus* spp.-based probiotics in cervical mucus the survival of *M. bovis* could be affected due to the acidification of the medium after LAB proliferation. In effect, *M. bovis* was negatively affected by the addition of L2 in cervical mucus after 15 h in incubation. That concurred with a significant reduction in pH from 8.4 to 5.6. Previous studies have reported the effectiveness of the medium acidification to control the growth of mycoplasmas. For instance, the acidification to pH 4 during 1 h was enough to eliminate viable *M. bovis* organisms in milk [23]. We also found out that the viability of *M. bovis* was negatively affected by the addition of L2 in diluted bull semen which coincided with a significant decrease of pH after 15 h in incubation [25]. Other authors observed a harmful effect on *Mycoplasma agalactiae* and *Mycoplasma mycoides* subsp. *capri* when diluting contaminated goat ejaculate in an acidic semen extender (pH ≤ 6) [24]. On the contrary, the abnormally elevated vaginal pH (> 4.5) in women with bacterial vaginosis may promote the survival of *Mycoplasma genitalium* [30]. On the other hand, the addition of L1 in cervical mucus did not have a negative impact on *M. bovis* survival. Although no significant LAB growth was observed, it must be noted that these bacteria remained viable in cervical mucus. Indeed, the significant decrease of pH from 8.40 to 6.73 in cervical mucus with *M. bovis* and L1 (condition 4) could be a consequence of the living bacteria metabolism.

The survival of *M. bovis* was not compromised in untreated cervical mucus whose concentration remained constant after 15 h in incubation. We believe that the cervical mucus composition could benefit the pathogen growth. As mycoplasmas have a small genome size, their metabolic activities are limited and they are largely dependent on external sources. Indeed, the cervical mucus soluble part contains basic elements for mycoplasmas growth and survival, including lipids such as cholesterol, proteins, amino acids, and carbohydrates [5, 31]. Besides, LAB were quantified in cervical mucus contaminated with *M. bovis* but without neither L1 nor L2. This was an expected result since previous authors have isolated LAB from the bovine vagina [17]. Other authors found that *Lactobacillus* spp. were prevalent among cows microbiota, although at low abundances, and they associated the near-neutral pH observed in the cow’s vagina with the low abundances of this bacterial genus [18].

The inoculums L1 and L2 were viable in cervical mucus without *M. bovis,* and a significant LAB growth was observed after 15 h in incubation. This concurred with a significant reduction in pH from 8.4 to 6.64 in condition with L1 and from 8.40 to 5.60 in condition with L2. Synergies occurring with the saprophytic cervical mucus flora or the presence of nutrients in this medium might promote the survival of L1 and L2 [5, 17, 18, 31]. Undoubtedly, this is an important finding from a preventive point of view because the administration of LAB 15 h before AI could reduce the vaginal pH and complicate the proliferation of semen contaminating bacteria. Nevertheless, at this point, we can only make assumptions that should be further investigated in living animals.

Overall, our results suggest that the vaginal administration of probiotics might be useful to control *M. bovis* proliferation in the cervico-vaginal tract of cows. This could be an interesting tool to minimize the risk of colonization that might occur after AI with contaminated bovine semen. On the other hand, the preventive administration of probiotics before insemination might create an inhospitable acid environment for the semen contaminating bacteria which could prevent their proliferation.

Notwithstanding such interesting results, the present assay has certain limitations: (i) Only PG45 was used for the experiments and therefore, additional *M. bovis* field isolates should be tested in the future. (ii) This is an in vitro study and our results may change in living animals. Developing an experimental model of vaginal contamination in those animals could be interesting to explore the impact of probiotics on *M. bovis* viability under in vivo conditions.

## Conclusions

Under the present in vitro conditions, we can confirm the susceptibility of the reference strain PG45 to the addition of *Lactobacillus* spp. at a concentration higher than 10^8^ CFU/mL in bovine cervical mucus. Our results suggest that the administration of probiotics via vaginal might be useful to control *M. bovis* proliferation in the cervico-vaginal tract of cows.

## Methods

### Animals and cervical mucus samples

Samples from ten different Holstein Friesian dairy cows between one and five deliveries, from a commercial farm in Murcia (Spain), were obtained for this assay on three different days from July to November of 2018. The first sampling was carried out in July and the second and the third samplings in November (with a time interval of 20 days). Five, three and two samples were respectively collected on day 1, 2 and 3 from healthy cows and free of abnormal genital discharges or anatomical abnormalities of the reproductive tract. The animals were immediately released after collecting the samples. The animals were randomly selected from a herd of approximately 800 cows officially free of tuberculosis, brucellosis and enzootic bovine leukosis, and without any other notifiable disease in Spain [32]. The herd had no history of clinical outbreaks associated with *M. bovis* in the past 10 years. No mycoplasmas were detected in the bulk tank milk during the year of the study.

An Ovsynch protocol [33] was used for heat and ovulation synchronization and the samples were obtained during estrus and before AI. For sample collection, two technicians were necessary. We first emptied the fecal contents of the rectum. Once free of feces, the vulva and the perineal region were washed with soap and water, rinsed with water, dried with sterile gauze swabs and disinfected with a chlorhexidine solution. The samples were obtained by transrectal massaging of the cranial cervix and placed in 100 ml sterile plastic bottles. The samples were immediately placed on ice in an isothermal box and transported to the laboratory within 1 h [34].

Each day, samples contaminated with feces were discarded and a pool of cervical mucus was obtained by mixing clean samples. Thereby, three samples on day 1, and two samples on days 2 and 3 were finally selected for the study.

Only cows free of cervical *M. bovis* were used in the present study. The absence of mycoplasmas in cervical mucus samples was confirmed by culture in mycoplasmas specific media and the presence of *M. bovis* was further discarded by polymerase chain reaction (PCR) [35].

### Preparation of modified SP4 medium

The medium SP4 was prepared following previous recommendations [36] but with some modifications. The modified medium is composed of three parts (A, B and C). Part A is composed of 4.2 g of Difco PPLO broth (BD), 6.4 g of Bacto Peptone (BD), 12 g of Bacto Tryptone (BD) and 724 mL of deionized water. The solid medium includes 7 g of European Bacteriological Agar (Conda-Pronadisa). The pH is adjusted to 7.8 and then part A is autoclaved at 121 °C for 20 min. Part B is composed of 60 mL of RPMI-1640 (Sigma-Aldrich), 21 mL of fresh yeast extract 50% w/v, 2.4 g of yeast extract (Conda-Pronadisa), 4.8 mL of phenol red 0.5%, (Sigma-Aldrich) and 0.642 g of ampicillin sodium salt (Fisher bioreagents). The pH is adjusted to 7.2 and then part B is filter sterilized through a 0.22 μL pore size filter. Part C is composed of 251 mL of heat-inactivated horse serum (Hyclone) for 30 min at 56 °C. Note that, in this study, the SP4 broth medium did not include ampicillin in part B.

### Preparation of the *M. bovis* and Lactobacillus spp. inoculums

An inoculum of *M. bovis* (1 × 10^9^ CFU/mL) and two inoculums of *Lactobacillus* spp., L1 (3.24 × 10^6^ CFU/mL) and L2 (3.24 × 10^8^ CFU/mL) were prepared in a modified broth SP4 as previously described [25]. The reference strain PG45, and the lyophilized commercial probiotic, NS Femibiotic®, based on a mix of *Lactobacillus crispatus*, *Lactobacillus gasseri* and *Lactobacillus brevis,* were employed for preparing the inoculums of *M. bovis* and L1 and L2 respectively.

### Experimental conditions

Seven in vitro conditions were experimentally prepared in cervical mucus and/or modified SP4 following an experimental protocol previously described [25].

Table 1 shows the volumes of cervical mucus, SP4, and *M. bovis* and *Lactobacillus* spp. inoculums that were used to elaborate each condition. Conditions 1, 2 and 3 were controls prepared to assess the viability of *M. bovis* and *Lactobacillus* spp. (L1 and L2) in cervical mucus. No controls were included in SP4 because the viability of these bacteria in this medium had been confirmed before the experiment. Conditions 6 and 7 were only included to verify if the viability of *M. bovis* would be affected by the addition of probiotic in a medium for mycoplasmas growing such as SP4.

The experimental conditions were carried out in eppendorf tubes: Firstly, cervical mucus or SP4 were added to the corresponding tubes followed by the inoculation with *M. bovis* and/or the inoculation with L1 or L2. The tubes were then incubated at 37 °C for 15 h (h15) and hour 0 (h0) was set after 15 min of incubation. Counts of *M. bovis* and LAB were made at 0 and 15 h. At h0, the counts were made to estimate the real concentration of bacteria present in each experimental condition at the beginning of the experiment. The next determination was performed at h15 to give the probiotic enough time for growing and acidifying the cervical mucus, and so any effect on *M. bovis* viability could be seen. Furthermore, pH measurements were made at both times.

Three replicates of the assay were conducted on different days (one replicate for each day of sample collection).

### Determination of *M. bovis* and LAB viability

Counts (CFU/mL) of *M. bovis* and LAB were conducted on SP4 and MRS agar following a protocol previously described [25]. The counts of *M. bovis* were only conducted in conditions contaminated with the mycoplasma (Table 1, *n* = 5) while the counts of LAB were determined in the seven conditions.

### Statistical analyses

Bacterial counts were converted to log (1 + C), being C the count of CFU/mL measured for each organism and analytical condition. Statistical analyses were conducted with a general linear procedure implemented in the program Statistical Analysis System (SAS) [37] according to the model: Y_ijk_ = μ + S_i_ + C_j_ + T_k_ + CT_jk_ + e_ijk_ where Y_ijk_ = pH and log CFU/ml of *M. bovis* and log CFU/mL of LAB (dependent variables); μ *=* mean; S_i_ = sample effect; C_j_ = effect of experimental conditions; T_k_ = effect of time; CT_jk_ = effect of the interaction experimental condition-time; e_ijk_ = residual effect.

## Data Availability

The datasets used and analyzed during the current study are available from the corresponding author on reasonable request.

## Abbreviations

AI: Artificial insemination
CFU: Colony-forming units
CM: Cervical mucus
LAB: Lactic acid bacteria
L1: *Lactobacillus* spp. at a concentration of 3.24 × 10^6^ CFU/ mL
L2: *Lactobacillus* spp. at a concentration of 3.24 × 10^8^CFU/ mL
MB: Mycoplasma bovis
PCR: Polymerase chain reaction
SAS: Statistical analysis system
SEM: Standard error of the mean
